# Rural access to MAT in Pennsylvania (RAMP): a hybrid implementation study protocol for medication assisted treatment adoption among rural primary care providers

**DOI:** 10.1186/s13722-019-0154-4

**Published:** 2019-08-01

**Authors:** Gerald Cochran, Evan S. Cole, Jack Warwick, Julie M. Donohue, Adam J. Gordon, Walid F. Gellad, Todd Bear, David Kelley, Ellen DiDomenico, Jan Pringle

**Affiliations:** 1 Program for Addiction Research, Clinical Care, Education, and Advocacy, University of Utah School of Medicine, 30 N. 1900 E, Salt Lake City, UT 84132 USA; 2 University of Pittsburgh Graduate School of Public Health, 130 De Soto St, Pittsburgh, PA 15261 USA; 3 University of Pittsburgh Program Evaluation Research Unit, 5631 Baum Blvd, Pittsburgh, PA 15206 USA; 4 University of Pittsburgh School of Medicine, 3550 Terrace St, Pittsburgh, PA 15213 USA; 5 Pennsylvania Department of Human Services, 625 Forster St, Harrisburg, PA 17120 USA; 6 University of Pittsburgh School of Pharmacy, 3501 Terrace St, Pittsburgh, PA 15213 USA

**Keywords:** Opioid use disorder, Medication assisted treatment, Rural, Implementation

## Abstract

**Background:**

The continued escalation of opioid use disorder (OUD) calls for heightened vigilance to implement evidence-based care across the US. Rural care providers and patients have limited resources, and a number of barriers exist that can impede necessary OUD treatment services. This paper reports the design and protocol of an implementation study seeking to advance availability of medication assisted treatment (MAT) for OUD in rural Pennsylvania counties for patients insured by Medicaid in primary care settings.

**Methods:**

This project was a hybrid implementation study. Within a chronic care model paradigm, we employed the Framework for Systems Transformation to implement the American Society for Addiction Medicine care model for the use of medications in the treatment of OUD. In partnership with state leadership, Medicaid managed care organizations, local care management professionals, the Universities of Pittsburgh and Utah, primary care providers (PCP), and patients; the project team worked within 23 rural Pennsylvania counties to engage, recruit, train, and collaborate to implement the OUD service model in PCP practices from 2016 to 2019. Formative measures included practice-level metrics to monitor project implementation, and outcome measures involved employing Medicaid claims and encounter data to assess changes in provider/patient-level OUD-related metrics, such as MAT provider supply, prevalence of OUD, and MAT utilization. Descriptive statistics and repeated measures regression analyses were used to assess changes across the study period.

**Discussion:**

There is an urgent need in the US to expand access to high quality, evidence-based OUD treatment—particularly in rural areas where capacity is limited for service delivery in order to improve patient health and protect lives. Importantly, this project leverages multiple partners to implement a theory- and practice-driven model of care for OUD. Results of this study will provide needed evidence in the field for appropriate methods for implementing MAT among a large number of rural primary care providers.

## Background

The high prevalence of opioid use disorder (OUD) in the US continues to have serious public health repercussions, including overdose death. Approximately 1.9 million individuals report a current prescription-related OUD [1], and the prevalence of heroin-related OUD has more than tripled in the past decade [2]. OUD is a chronic health condition that requires long-term multimodal clinical approaches to engage, treat, retain, promote long term recovery, and prevent additional opioid-related adverse events among patients, such as overdose [3]. OUD in rural areas has taken a particularly heavy toll due to the dearth of health and human services resources available within these communities [4, 5]. Across the US, there likewise has been a continued shift in the cause of overdose deaths in the last 20 years from prescribed opioids, to heroin, to synthetic opioids [6]. Given these rural health challenges, it is paramount to leverage existing resources and strengthen rural service systems to meet the needs of those with OUD.

One area for potential rapid expansion of opioid addiction services is office-based prescribing of medication assisted treatment (MAT), primarily buprenorphine and buprenorphine/naloxone (hereafter defined as buprenorphine) and extended-release naltrexone in combination with psychosocial counseling services. Although office-based treatments, such as buprenorphine, have been shown to be effective in OUD treatment, recent data show 56% percent of rural counties in the US lack a buprenorphine prescriber, and 30% of rural residents in the US live in a county without a buprenorphine prescriber [7]. Rural physician practices report a number of challenges with implementing buprenorphine prescribing. These challenges include concerns of buprenorphine diversion, lack of behavioral health providers/services to which patients can be referred, time constraints to learn about and to provide MAT services, lack of clinic staff to support patient needs, and lack of sufficient financial support/reimbursement for MAT services [8]. In addition, providers also report stigma as a critical barrier faced in prescribing buprenorphine for OUD in rural areas [8]. Given the need for rapid response to the opioid epidemic while considering the challenges of providing high quality office-based MAT, thoughtful and concerted efforts for expanding access to MAT in rural areas are crucial.

### Expanding MAT through primary care

Management of patients with chronic health conditions requires intentional efforts to target system- and patient-level factors necessary to significantly and clinically improve patient outcomes [9–13]. The Chronic Care Model importantly is recognized as an effective paradigm for the long term management of chronic conditions and has received attention regarding management of substance use disorder (SUD) [14, 15]. Building upon principles of chronic care management, the purpose of this article is to describe the protocol of a study working to expand access to MAT by utilizing an implementation science framework to initiate a MAT service model in 23 rural Pennsylvania (PA) counties. This project was among a series of initiatives funded by the Agency for Healthcare Research and Quality to expand access and quality of MAT in rural settings (AHRQ 5R18HS025072, PI: DiDomenico). The methods described in this paper have the potential to aid in the advancement of the current implementation science knowledgebase for expanding access to MAT services for OUD in rural areas of the US.

## Methods

### Study design and population

**Fig. 1:**
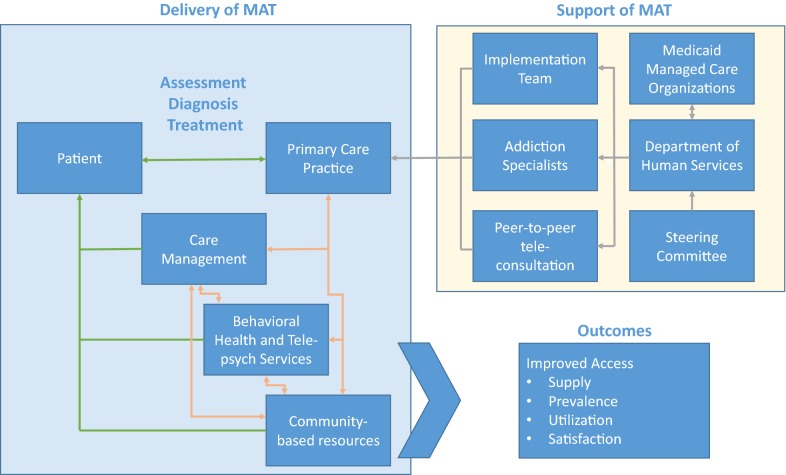
Rural access to MAT in pennsylvania (RAMP) model diagram

This project is a hybrid implementation study that is specifically recruiting, training, and supporting rural primary care providers (PCPs) in delivering MAT services to Medicaid enrollees served within their practices. Hybrid designs focus on assessing formative study outcomes related to implementation and summative outcomes related to intervention efficacy. This project leverages existing payers and local providers in identified rural communities to support MAT expansion in order to facilitate long-term service provision to patients with OUD (Fig. 1). Individuals enrolled in Medicaid living in rural areas is a critical population of focus given that the Medicaid program provides health coverage to 24% of non-elderly adults in rural areas. Moreover, the Medicaid program provides health coverage for nearly 40% of those with OUD in the US [16].

### Setting

This project is currently being implemented among practices that serve Medicaid enrollees in 23 PA counties designated as rural by Rural–Urban Continuum Codes [17, 18]. Pennsylvania is 8th overall in overdose deaths within the US [19]. The counties in this project were selected based on whether their OUD prevalence and/or overdose death rate was above the national average [20], and thus, represented areas of greatest need for OUD treatment expansion.

### Project team

This project is being executed through collaboration among a number of key state/private organizations, leaders, researchers, and stakeholders. The PA Department of Human Services (DHS), which is the state agency that administers the Medicaid program and is a large payer of MAT of the managed care Medicaid providers targeted in this project. DHS collaborates with the PA Department of Drug and Alcohol Programs and the following agencies to form a Steering Committee that guides the project:

- PA Medicaid Managed Care Organizations (MCOs).
- An Implementation Team located within the University of Pittsburgh School of Pharmacy.
- Addiction medicine clinical and educational programs within the UPMC Western Psychiatric Institute and Clinic and the University of Utah.
- Local care management professionals.
- A Project Evaluation Team based at the University of Pittsburgh and University of Utah; comprised of addiction medicine, internal medicine, health policy, and social work faculty and staff.
- Participating primary care practices and the patients which they serve.

### Partner roles, site identification, and recruitment

PA DHS is facilitating all partnerships with the participating project collaborators and is managing all support components of the intervention for the participating primary care practices. PA DHS is also receiving active support and feedback from the project Steering Committee. PA DHS, Medicaid MCOs, and the Implementation Team work together to identify, recruit, and engage primary care practices. MCOs also specifically recommend practices to DHS based on their knowledge of their existing capabilities, Medicaid enrollee patient volume, and likelihood of successfully initiating and sustaining MAT. Once MCOs provide recommendations to DHS; the DHS team, MCO representatives, and the Implementation Team begin the recruitment and engagement process. The Implementation Team also has engaged in a number of additional outreach activities to contact potential practices, including directly contacting clinics, rural-based health systems, healthcare agencies, and provider associations as well as seeking referrals from behavioral health providers in the area. The goal is to recruit 24 practices within the 23 counties during the three project years.

### Practice-based MAT delivery model intervention

The model of MAT implemented in all participating practices adheres to current guidelines from the American Society of Addiction Medicine (ASAM) on the use of medications in the treatment of OUD [21, 22]. The ASAM delivery model focuses on provider processes that include assessment, diagnosis, treatment, and considerations for populations with behavioral health needs.

#### Screening, care management referral, and assessment

Participating practices are trained in and subsequently screen patients for OUD using the NIDA-Modified Alcohol, Smoking, and Substance Involvement Screening Test [23]. Primary care practices are also trained in the provision of motivational interviewing principles for the purpose of engaging patients and motivating them to accept a referral to the Rural Access to MAT in Pennsylvania (RAMP) program. Patients that screen positive on the screening test are then referred to RAMP-involved care managers who conduct a confirmatory assessment for OUD using the *Diagnostic and Statistical Manual of Mental Disorders* (DSM-5) [24] criteria and who also assess patients’ other mental health and psychosocial needs. From this assessment, patient eligibility for MAT is determined and the patient is enrolled with RAMP. Following enrollment, patients are given a comprehensive assessment, which includes a physical exam, screening for concomitant medical conditions, appropriate laboratory testing, pregnancy testing, and tobacco screening and cessation counseling. Following assessment, PCPs diagnose patients with OUD if confirmation is obtained via: (1) patient’s reported substance use behaviors during the confirmatory assessment, (2) communication with an ancillary prescribing provider, or (3) through a validated clinical scale (such as the Clinical Opioid Withdrawal Scale). Urine testing is also recommended whenever possible and deemed appropriate to help confirm patient histories.

Patients are subsequently referred and provided with a warm handoff by the PCP and/or clinic staff using principles of motivational interviewing to care management professionals from organizations that participate in DHS’ OUD Centers of Excellence programs [25], county public SUD treatment management entities, local substance use treatment centers, or onsite nurse care managers who have volunteered to be trained by the Implementation Team in the Massachusetts nurse care manager model [26]. DHS Centers of Excellence are a network of sites across PA that receive state support to provide OUD services to patients and support to health care providers who are caring for those with OUD [25]; Centers are specifically modeled after the behavioral health home model [27–29]. Care managers further assess the patient to determine the level of needed OUD care (e.g., whether patients require ambulatory detoxification). Assessment to determine the level of OUD care follows either the Pennsylvania Placement Criteria for Adults [30] or the *ASAM Criteria* [31]. Once the detoxification is completed or determined to not be needed, the care management professional and patient make a subsequent appointment with the PCP for MAT treatment recommendation and initiation.

#### Treatment recommendation and induction

PCPs and the care management team consider patient preferences and treatment history when determining the optimal treatment recommendations and treatment settings for the patient, which focus in the current project is buprenorphine or extended-release naltrexone delivered in outpatient settings. However, if the PCP believes that after consultation with intervention supports (e.g., addiction medicine teleconsultation), the patient is best suited for a higher-level of care (e.g., inpatient treatment), the PCP makes such a referral in coordination with the care management professionals.

As part of recruitment of the implementation sites, we have found that not all participating PCPs are immediately comfortable with prescribing all types of medications for OUD. The Implementation Team assesses where each site is at with their readiness to provide MAT, and some practices begin by screening and referring, such as a “hub and spoke” model, and others pursue their DATA 2000 waiver to begin prescribing buprenorphine. However, the Implementation Team continues to work with PCPs with the ultimate goal of increasing their experience and comfort with offering MAT.

#### Care management and psychosocial services

In concert with MAT initiation, care management professionals coordinate a number of different services to support the patient’s OUD treatment. These services include additional community behavioral health services, continuing patient engagement in OUD treatment, assisting patients navigating transitions of care (e.g., inpatient hospitalization to outpatient treatment), patient self-management training, peer support, supporting patients in ongoing behavioral health and OUD counseling services, and communication with law enforcement, such as parole or probation officials. Importantly, care management also involves connecting patients to community-based services and recovery support specialists to help them address housing, food security, or transportation needs.

#### PCP clinical support

As rural practices are enrolled in the project, they are offered clinical supports to ensure successful MAT service provision: which include peer consultation, peer support, and education. PCPs have access to a peer-to-peer teleconsultation service staffed by addiction medicine providers or that are facilitated by the Implementation Team. PCPs can contact specialists to receive consultation on treatment decisions for individual patients and discuss questions or concerns about the structure of MAT in their clinic. PCPs also have the option of obtaining consultation services via email. The project goal for responding to consultation requests is to return all provider phone queries within 30 min and email within 24 h. Teleconsultation addiction experts can also coordinate with the program website to communicate commonly asked questions and concerns.

Clinical addiction specialists and the Implementation Team offers site-specific tailored intervention resources designed to improve and sustain access to MAT for OUD in primary care settings. Provision of such resources that are aimed at increasing the knowledgebase and clinical proficiency of prescribers and multidisciplinary providers have shown to improve the number of prescribers of MAT and to encourage and enhance high quality care [32, 33]. In the current project, the Implementation Team, project website, and consulting addiction specialist offer MAT training and education for each clinic site, a link to the DATA2000 waiver training for PCPs needing certification, a webinar series based on clinic questions and concerns or other related topics identified as needed by project staff, training and support for patient self-management, and consultation services.

#### Financial support

Noted above, rural primary care practices often lack the resources necessary to provide MAT. All MAT, behavioral, and physical health related services delivered within the project service model are reimbursable through Medicaid MCO contracts with providers. Practices recruited to participate in this project are directly connected with billing specialists from their corresponding Medicaid MCOs to instruct on appropriate procedures and available billing codes to ensure adequate reimbursement for services provided to sustain MAT expansion within the clinic.

### Implementation process

The process for supporting the MAT program implementation follows the Framework for Systems Transformation (STF) [34, 35] and encompasses 7 domains, see Table 1. The domains are: Vision, Leadership, Performance Measurement, Internal Learning, External Learning, Organizational Culture/Behavior, and Organizational Structure. The implementation team uses the STF to understand the organizational health of each implementation site and partnership, which helps determine the amount of resources necessary to ensure the program meets its implementation goals and to support the sites in the development of a common programmatic vision, specification of implementation processes, collection of implementation related data, and application of continuous quality improvement efforts.

**Table 1:** Framework for systems transformation and RAMP project activities undertaken by implementation team

Vision	Leadership	Performance measurement	Internal learning	External learning	Organizational culture/behavior	Organizational structure
Develop site-specific vision statement that interfaces with RAMP project implementation vision statement, e.g.: [Name of Primary Care Practice] will increase patient access to MAT and addiction specialty services in [the community] by providing the highest quality MAT services to our patients who suffer from opioid use disorder	Identify system/site decision makers for collaboration and engagement Identify champions to support implementation at each site Provide ongoing support to site/system leaders throughout the implementation process	Develop core set of data components for primary care, care management, and others to collect in the course of delivering the project activities Assist sites in collection of data components, tailoring methods to sites’ capabilities Clean, verify, and report back aggregated data to sites for performance improvement planning	Employ Lean principles to support sites to improve implementation Employ Lean Rules in Use to ensure implementation process/roles are accurately specified Update/improve performance management reports continuously to ensure understanding and identification of needed changes Assign/monitor performance benchmarks to metrics to provide sites and RAMP team targets for implementation efforts	Develop curriculum and training to provide skills/resources to physicians, advanced practice professionals, care management staff, and other involved staff that these professionals and the Implementation Team identify as important Update/modify curriculum and training topics based on site requests/needs, including attainment buprenorphine prescribing waivers	Perform brief organizational health assessments of systems and sites to determine level of implementation difficulty in order to anticipate barriers and required resources to support implementation	Facilitate primary care sites to participate in 1 of 4 MAT models to enhance site engagement/sustainability, which include (1) Site performs all aspects of MAT and patient monitoring (2) Site performs all aspects of MAT, and patient monitoring is referred to community partners (3) Site screens patients for potential MAT need and refer patients to “hubs” for induction and monitoring (4) Site screens patient for MAT need and refers to “hub” for induction, monitoring, and primary care services Implementation model involves “concierge technical assistance,” i.e., ongoing quasi real-time individualized assistance aimed at providing sites what they need when they need it determined via regular communication

*RAMP* rural access to MAT in Pennsylvania, *MAT* medication assisted treatment

### Quality improvement efforts

Data collected from each participating site are used to ensure that the primary care practice and collaborating partners are implementing the Project RAMP as designed. The data collected from the primary care practices includes SUD screening counts and data related to naltrexone and buprenorphine administration, such as the stage of treatment (initiation versus maintenance) and date of administration. Other collaborating partners, such as the care management teams, collect data related to contact and follow-up with patients, assessment results, and reasons for discharge from treatment. This data is provided via a web-based application system and/or paper-based spreadsheets faxed to the implementation team, which regularly provides aggregated data back to the sites and to the Steering Committee.

### Outcome evaluation

To assess outcomes, the Evaluation Team will conduct a retrospective claims-based analysis and a prospective patient-level survey. For the claims-based analysis, the Evaluation Team will use Pennsylvania Medicaid enrollment, claims, and encounter data to assess the extent MAT utilization increased for Medicaid beneficiaries in participating counties and practices following the intervention implementation during the three-year project period, 2016–2018. Using these data, a number of dependent variables will be constructed for analysis (Table 2), for instance: MAT provider supply defined as number of unique providers billing Medicaid for MAT, OUD prevalence, and MAT utilization. With these indicators, outcomes will be examined across time to assess whether implementation is correlated with changes in the indicators.

**Table 2:** Claims-based measures and definitions for study outcomes

Measure	Operational definition
*Supply measures*	
Percent of physicians certified to prescribe buprenorphine	Number of physicians on SAMHSA list of buprenorphine or naltrexone prescribers/# Medicaid-participating physicians in county
Percent of primary care practices delivering MAT	Number of physicians with any prescribing of buprenorphine or naltrexone/# Medicaid-participating physicians in county measured in claims data
*Prevalence measures*	
OUD diagnoses	OUD ICD-9/ICD-10 code on inpatient, outpatient or professional claims
Overdose events	Inpatient stays or ED visits with an opioid overdose ICD-9/ICD-10 code
OUD diagnoses among those with evidence of misuse of prescription opioids	Rate of OUD diagnosis among those with opioid misuse using an algorithm from Sullivan et al. [46] that uses # prescribers, # pharmacies, and # days short- and long-acting opioids
*Utilization measures*	
Use of MAT	Any prescription fill for buprenorphine or IM naltrexone among those with OUD
Use of psychosocial supports	Any visit with a service code for psychosocial support for OUD
Access to counseling for OUD	Any visit with a service code for counseling regarding psychosocial and pharmacologic treatment options among those with OUD
Duration of MAT	Number of months with proportion of days covered > 80%
Access to tele-psychiatry	Any visit with a service code for a tele-psychiatry visit
*Outcome measures*	
Likelihood of any and SUD or mental health-related emergency department visit	SUD or mental health ICD-9/ICD-10 code as primary diagnosis for an emergency department visit
Likelihood of any and SUD or mental health-related inpatient hospitalization	SUD or mental health ICD-9/ICD-10 code as primary diagnosis for an inpatient hospitalization

*SAMHSA* Substance Abuse and Mental Health Services Administration, *MAT* medication assisted treatment, *OUD* opioid use disorder, *ICD* international classification of diseases, *SUD* substance use disorder

For the prospective patient survey, participants will be recruited at the various clinic sites. The purpose of the survey will be to assess patient’s perceived access to care and level of satisfaction as well as changes in these domains over a 6-month follow up period after patients are recruited into the project. Recruitment of patients is conducted by both the primary care physician and the care management team. Patient survey packets are distributed to eligible individuals, and those interested are asked to mail back a form indicating their willingness to be contacted by the research team. Survey responses cover items from the Consumer Assessment of Healthcare Providers and Systems survey, Primary Care Center Study Patient Satisfaction Questionnaire, and the Client Perception of Coordination Questionnaire. In addition to close-ended survey questions, participants will also be asked a series of open-ended research questions, which will explore perceived satisfaction, quality of care, and stigma. Participants receive a $10 gift card as remuneration for participation.

## Discussion

This project provides a needed response to a major gap in the availability of MAT in rural areas. Results of this project have the potential to advance the field in two important ways. First, this project recognizes opioid-related adverse events, including OUD, are indeed chronic health conditions [3] and delivers MAT following a chronic condition management model of care, which models have demonstrated in previous research to result in important improvements for patient outcomes [9–13]. Specifically, the service model in this project initiates a redesign of the current delivery system into a proactive approach that facilitates care the management professionals working with rural PCPs, so patients may have access to an interdisciplinary team. This project also works with key staff to ensure leadership is invested and motivated to provide the care required for MAT and reimbursement is available to practices, which engagement efforts have been identified as critical aspects for sustainment of new practices within health care settings [36–38]. Efforts within Project RAMP also include providing expert-informed decision support to guide patient care and grow PCPs’ skillsets [39]. Lastly, Project RAMP offers training and expertise to PCPs via webinars and phone and email responses to questions on MAT and patient self-management. In the spirit of self-management, PCPs and care management professionals work to identify and refer patients to beneficial services and resources.

The second important contribution this project makes is that it follows an implementation model, the Framework for Systems Transformation [34, 35], in order to ensure high fidelity for model implementation. Given the gap between real-world public health application and scientific discovery in the field, rigorous monitoring and evaluation of implementation efforts of evidence-based health care models into practice has been increasingly valued in recent years [40–42]. Thus, the chronic care based MAT model of service utilized in this project follows an implementation process designed to iteratively monitor and provide feedback to project leaders and practitioners. Utilization of implementation science has broad support for evidence-based practice implementation and results in service adoption, maintenance, and long-term outcomes [40, 43–45]. Utilization of such a framework is especially important in rural areas given limited resources available for training and monitoring health and human services.

## Conclusion

Rural areas in the US have been hit particularly hard by the current OUD and overdose epidemic given the paucity of health and human services resources available [4, 5]. This project has the potential to provide a model for how other states in the US can expand access to MAT. Components of this project may be transferable to other rural counties in the US, including working with payers to identify communities in need, recruiting key staff within PCP practices to help move forward project needs, and leveraging existing community resources to support behavioral and care management needs of patients.

## Data Availability

Data sharing is not applicable to this article as no datasets were generated or analyzed during the current study—data are forthcoming.
